# Polyphenolic Extracts of Edible Flowers Incorporated onto Atelocollagen Matrices and Their Effect on Cell Viability

**DOI:** 10.3390/molecules181113435

**Published:** 2013-10-30

**Authors:** Jorge López-García, Zdenka Kuceková, Petr Humpolíček, Jiři Mlček, Petr Sáha

**Affiliations:** 1 Centre of Polymer Systems, Tomas Bata University in Zlín, nám. T.G.Masaryka-5555, Zlín 76001, Czech Republic; E-Mails: vextropk@gmail.com (J.L.-G.); kucekova@ft.utb.cz (Z.K.); saha@utb.cz (P.S.); 2 Polymer Centre, Faculty of Technology, Tomas Bata University in Zlin, T.G.M. sq. 275, Zlin 76272, Czech Republic; 3 Department of Food Analysis and Chemistry, Faculty of Technology, Tomas Bata University in Zlin, nám. T.G.Masaryka-5555, Zlin 76272, Czech Republic; E-Mail: mlcek@ft.utb.cz

**Keywords:** edible flowers, polyphenolic extracts, atelocollagen thin films, cell viability, tissue engineering

## Abstract

The phenolic extract of chives flowers (*Allium schoenoprasum*, *Liliaceae*), introduced Sage (*Salvia pratensis*, *Lamiaceae*), European elderberry (*Sambucus nigra*, *Caprifoliaceae*) and common dandelion (*Taraxacum officinale,* *Asteraceae*) were characterised by High Performance Liquid Chromatography and incorporated in different concentrations onto atelocollagen thin films. In order to assess the biological impact of these phenolic compounds on cell viability, human immortalised non-tumorigenic keratinocyte cell line was seeded on the thin films and cell proliferation was determined by using an MTT assay. In addition, their antimicrobial activity was estimated by using an agar diffusion test. Data indicated the concomitance between cell viability and concentration of polyphenols. These findings suggest that these phenolic-endowed atelocollagen films might be suitable for tissue engineering applications, on account of the combined activity of polyphenols and collagen.

## 1. Introduction

The overriding function of tissue engineering is to design biological substitutes for skin/organ replacement. Within this framework, collagen is an abundant mammalian protein which is involved in many important biological functions, such as tissue formation, cell attachment and proliferation. This protein is employed in diverse fields, including medicines, foods, cosmetics and tissue engineering. It is deemed as a primary source in biomaterial applications and one of the most useful biomaterials [1,2,3,4]. The current medical applications of collagen as a biodegradable material are associated with its natural properties, including low immune response and the ability to promote cell growth [5].

Skin comprises essentially three types of cell: keratinocytes, melanocytes and fibroblasts. It is foreseen through wound healing, transplantation and cell culture studies that HaCaT cells may be used as an *in vitro* model for highly proliferative epidermis in tissue engineering. HaCaT cell line is spontaneously transformed into human keratinocytes which have the traits of basal epidermal keratinocytes. Hence, this cell line may be exploited as an *in vitro* model for highly proliferative epidermis. In fact, several wounds have been successfully re-surfaced by culturing autogenic keratinocytes cells [6,7,8].

The extensive uses of chives (*Allium schoenoprasum*), introduced sage (*Salvia pratensis*, *Lamiaceae*), European elderberry (*Sambucus nigra*, *Caprifoliaceae*) and common dandelion (*Taraxacum officinale*) range from culinary for flavouring dishes to medical purposes, such as antitussives, antiseptics, antifungals, antispasmodics, and anti-inflammatories, amongst other properties [9,10,11,12]. These pharmaceutical uses are associated with the rich amount of polyphenols which are found in these plants [13,14]. It is well established that polyphenols have antitumoral properties by virtue of their antioxidant activities, which have been studied for many years [15,16,17,18]. The polyphenols content and concentration vary according to each plant, time of the year, and age of the plant, amongst other kinds of factors [19,20,21,22,23].

Acute care is a serious concern in tissue replacement. Several implants have to be removed by their poor performances. Indeed, infections, mycoses and inflammations are common causes of biomaterial implant failure in medicine and by extension, a threat to patients’ lives and a source of high costs [24,25].

Therefore, the main purpose of this contribution is aimed at the addition of the abovementioned edible flowers extracts onto collagen matrices and to evaluate the effect of their polyphenolic compounds on keratinocyte cell response and their antibacterial properties by means of cell viability and antimicrobial studies. In terms of novelty, to the best of our knowledge, it is the first time that those extracts are incorporated onto any kind of collagen matrix. The findings of this research seek to shed more light on fields, such as anti-infective biopolymers, phytochemistry, human cell growth and tissue engineering.

## 2. Results and Discussion

Table 1 summarises the compounds identified in the examined edible flowers along with their concentrations as detected by HPLC. Ferulic, caffeic and sinapic acid were found as the major constituents of the methanolic extracts of chives flowers, European elderberry and common dandelion respectively. Likewise, gallic acid was found in all the extracts.

**Table 1:** Phenolic constituents identified by HPLC. The content of the detected phenolic compounds is expressed as μg of compound/g of dry matter.

Name (compound number)	Wild Chive	Introduced Sage	European Elderberry	Common Dandelion
	*A*. *schoenoprasum*	*S*. *pratensis*	*S*. *nigra*	*T*. *officinale*
Gallic acid (1)	201.76	22.67	176.61	441.40
Coumaric acid (2)	207.29	/	/	/
Ferulic acid (3)	887.44	/	/	/
Rutin (4)	20.26	/	/	18.66
Resveratrol (50	/	/	/	274.92
Vanillic acid (6)	/	/	299.38	82.88
Sinapic acid (7)	/	/	/	593.04
Catechin (8)	/	37.56	/	/
Caffeic acid (9)	/	/	913.19	/
Total phenolic content (mg/g)	17.50	17.10	18.40	17.20

From these compounds, four (ferulic, sinapic coumaric and caffeic acid) belong to the hydroxycinnamic acids family, two (rutin and catechin) are types of flavonoids, one (vanillic acid) is a dihydroxybenzoic acid, one (gallic) a trihydroxybenzoic acid, and resveratrol is a stilbenoid. All of these phenols come from the shikimate and phenylpropanoids metabolic pathways. Indeed, phenylpropanoids are the largest group of secondary metabolites produced by plants mainly to counteract biotic or abiotic stresses, where the protective action is related to their antioxidant and free radical scavenging properties. For instance, the found flavonoids have shown a diversity of biological activities including antioxidant, antimicrobial, anti-inflammatory, antithrombosis, antihyper-cholesterolemic, anticancer activities, and spermatozoa activating bioactivity [26,27]. Ferulic acid has anti-inflammatory and antitumoral properties [28]. Gallic acid induces apoptosis in a series of cancer cell lines, and produces selective cytotoxicity against tumour cells with higher sensitivity than normal cells [29]. There is a plethora of information about the health benefits of consuming moderate amounts of red wine, which contains significant amounts of resveratrol [30,31,32]. Surprisingly, the flavonol quercetin, which is one of the most common flavonoids in plants, was not detected in any of these methanolic extracts. However, rutin, the glycoside formed between quercetin and the disaccharide rutinose was found [33]. It is important to emphasise that this study only examined 10 types of polyphenols and assuredly, there is a sort of undetected components in the extracts. For instance, volatiles, terpenoids, coumarins, chalcones, anthocyanins, that may be detected by gas chromatography, by using other HPLC column with a different polarity or a different solvent gradient [34,35,36,37,38].

**Figure 1:**
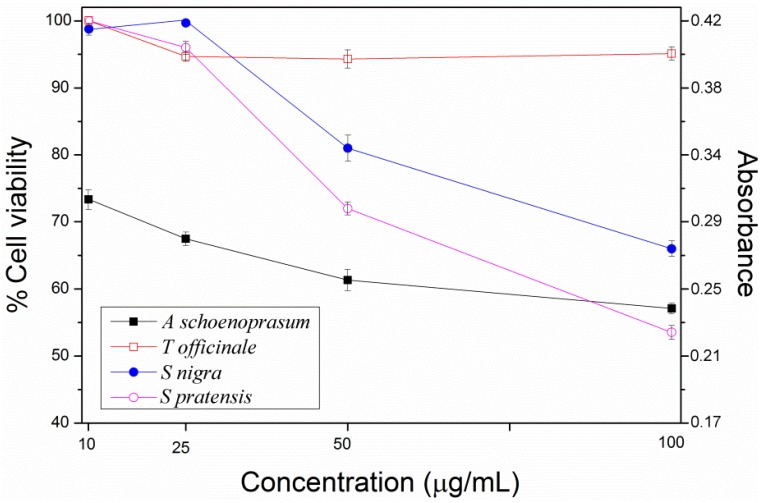
Cell viability curves with respect to concentration of the phenolic extracts incorporated onto the atelocollagen films. They illustrate the connexion between cell viability and amount of agent applied (the error bars signify standard deviation).

The effect of distinct concentrations of the above studied phenolic extracts on keratinocytes proliferation is depicted in Figure 1. The dependence of cell viability on concentration is clearly noticed. In general all the plots have the same pattern, where it shows a fall in cell viability by rising concentration, and the antiproliferative activity depends upon each particular herb.

Common dandelion (*Taraxacum officinale*) has a minor effect on cell viability with the highest cell proliferation rates. This curve exhibits a small drop followed by a plateau towards the end, where cell viability is around 95%. In contrast, chives flowers (*Allium schoenoprasum*) show the lowest rates (highest antiproliferative activity 72%–57%) describing a downwards trend. Introduced sage (*Salvia pratensis*) and European elderberry (*Sambucus nigra*) display similar behaviours and their graphs evince a slight decrease at 10 and 25 μg/mL, followed by a steep decline at the higher concentrations (50 and 100 μg/mL). According to ISO 10993-5, percentages of cell viability above 80% are considered as non-cytotoxic; within 80%–60% weak; 60%–40% moderate and below 40% strong cytotoxicity, respectively [39]. Thus, all the studied concentration of *Allium schoenoprasum*, 50 and 100 μg/mL of *Salvia pratensis*, and the highest concentration of *Sambucus nigra* possess weak toxicity to the keratinocytes cell line cultivated on the prepared atelocollagen films. The rest of the systems are innoxious to HaCaT cells. Pristine atelocollagen films were taken as the ones with 100% viability.

The antiproliferative activity of each extract may be related to the polyphenolic content differences, along with synergistic effects. For example, it has been substantiated that flavonoids have roughly higher reactivity than phenolic acids. Likewise, the combination of different subclasses of phytochemicals usually shows greater influence as a group than as individual entities. This might explain why the phenolic extract of *Salvia pratensis*, with only two detected compounds (the flavanol catechin and gallic acid) and *Allium schoenoprasum* with 20.26 μg/g of rutin and two hydroxycinnamic compounds, couramic and ferulic acid have the highest antiproliferative rates. In fact coumaric and ferulic acid together are efficient modulators of NF-κB activity compared with their effect separately [40,41,42]. Catechin and gallic acid individually have more reactivity than the rest of the phenolic counterparts identified here [43]. Gallic acid has three hydroxyls on its phenyl ring and catechin two hydroxyls on the B ring of its flavonoid backbone. Generally, for benzoic and phenylpropanoids, an increase in the number of hydroxyl groups results in a higher antioxidant activity. Compounds with two or three hydroxyl groups on the phenyl ring of phenolic acids or on the B ring of flavonoids present high antioxidant activity. The loss of one hydroxyl group represents a slight decrease of their activity, but the loss of two hydroxyl groups significantly diminishes it [44].

**Figure 2:**
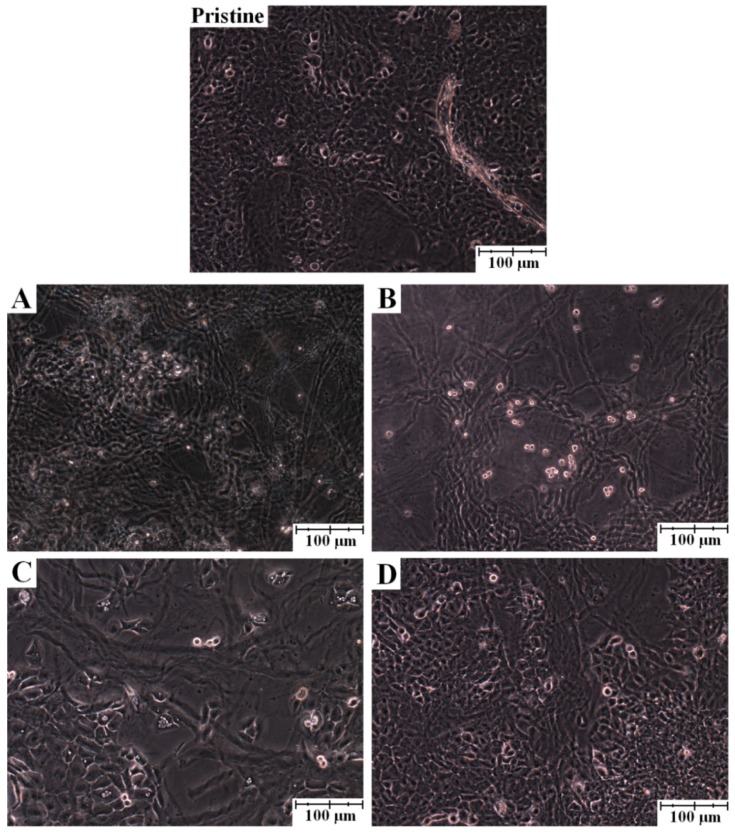
Light micrographs of human skin HaCaT keratinocytes in culture on atelocollagen thin films with 100 μg/mL phenolic extract of: (**A**) *Allium schoenoprasum*; (**B**) *Salvia pratensis*; (**C**) *Sambucus nigra* and (**D**) *Taraxacum officinale*.

As far as cell morphology is concerned, Figure 2 reveals the HaCaT cell growth on atelocollagen films with and without phenolic extracts, which shows cell aggregates in form of ripple-like areas adhered on the film surfaces. It may be also observed that some group of cells do not have the typical keratinocyte cell shapes, an anomaly that is probably a consequence of cell damage caused by the high concentration of phenols in the atelocollagen matrix [45,46].

Cell stability is a pivotal issue in cell culture. In that respect, it has been demonstrated that after four days in culture, a proper concentration of these polyphenolic extracts does not negatively influence cell viability.

Statistically speaking, the selected concentrations of *Allium schoenoprasum* as well as 50 and 100 μg/mL of *Salvia pratensis* and *Sambucus nigra* were found very statistically significant (99%, *p <* 0.01), whilst in the rest of the samples, the differences are not statistically significant compared with pristine atelocollagen thin films.

None of the added phenolic extracts evidenced any antimicrobial activity against pathogenic Gram-negative or Gram-positive bacterial strains. Nevertheless, it has been comprehensively demosntrated that polyphenols, such as catechin, gallic acid, ferulic acid, coumaric acid and resveratrol may act either alone or in mixtures as long-term anti-inflammatory, antifungal and antineoplastic agents, which are three of the most serious concerns in current medicine [47,48,49].

## 3. Experimental

### 3.1. Materials

Collagen gel from bovine splits (pH 5.2), which contains 16.2% of atelocollagen was supplied by Vipo A.S, Partizánske, Slovakia. Acetic acid 99% was obtained from Penta, Prague, Czech Republic. Tissue culture dishes of 40 mm diameter and individual wells of 96-well were commercially acquired from TPP, Trasadingen, Switzerland. A Vybrant^®^ MTT cell proliferation Assay kit V-13154 was purchased from Invitrogen Corporation (Carlsbad, CA, USA).

### 3.2. Extraction Conditions

Polyphenols were extracted from the following flowers: chives (*Allium schoenoprasum*, *Liliaceae*), introduced sage (*Salvia pratensis*, *Lamiaceae*), European elderberry (*Sambucus nigra*, *Caprifoliaceae*) and common dandelion (*Taraxacum officinale*). All flowers were botanically identified and picked from the White Carpathian Mountains, Zlin Region, Czech Republic and sent to the laboratory in the city of Zlin. Immediately after cutting the flowers, those were frozen and stored at −40 °C. The frozen herbs were homogenised in 90% methanol extracted at 4 °C for 30 min and subsequently centrifugated at 1990 rpm for 10 min to separate the supernatant. Sediments were subjected to a new extraction, which was repeated thrice. The extracts were carefully concentrated by using a Laborota 4011 digital rotary evaporator (Heidolph, Schwabach, Germany). The final concentration of each extract was adjusted to 1,000 mg/mL.

### 3.3. Determination of Polyphenols

The quantification of the total flower phenolic content was ascertained by using the Folin-Ciocalteu Assay. Extract (1 mL) was added to a 25 mL volumetric flask containing deionised water (20 mL) and later on Folin-Ciocalteu’s phenol reagent (1 mL, Sigma-Aldrich, St. Louis, MO, USA), was incorporated to the mixture. Three minutes after that, 20% Na_2_CO_3_ (5 mL) was added into the flask. The aqueous solution was mixed and completed to a final volume of 50 mL. After 30 min, the colour intensity was measured by using an UV-Mini 1240 spectrophotometer (Shimadzu, Kyoto, Japan) at 700 nm and compared to a no-tannin control sample. All samples were analysed in duplicate.

### 3.4. High Performance Liquid Chromatography (HPLC)

The determination of each polyphenol was carried out by using a Dionex UltiMate 3000 High-performance Liquid Chromatography system (Dionex, Sunnyvale, CA, USA). The HPLC was equipped with a Supelcosil LC-18-DB column (25 cm × 4.6 mm I.D. S-5 μm) employing a binary gradient of (A) 5% (v/v) acetonitrile, 0.035% (v/v) trifluoroacetic acid and (B) 50% (v/v) acetonitrile, 0.025% (v/v) trifluoroacetic acid. The flow-rate was set at 1.0 mL/min. The gradient elution profile began with A–B (90:10), and then B was gradually increased to 20% at 10 min, to 40% at 16 min, to 50% at 20 min and back to 40% from 25 to 27 min.

### 3.5. Atelocollagen Thin Films Preparation

The atelocollagen was solubilised in 0.1 M acetic acid to prepare a 0.1% *w/w* solution using an IKA RCT stirring machine (IKA^®^ works, Inc., Staufen, Germany) for 1 h at 1000 rpm. Then, 2 mL of this solution was casted on tissue culture dishes. The methanolic extracts were diluted to obtain final concentrations of 100, 50, 25 and 10 μg/mL and incorporated into the casted solutions. The solvents (acetic acid and methanol) were evaporated at ambient conditions for three days. Thin films of pristine atelocollagen were prepared and set as experimental blanks.

### 3.6. HaCaT Cell Incubation

Human immortalised non-tumorigenic keratinocyte cell line HaCaT, (skin tissue, Caucasian ethnicity; 62 years of age, male gender) was supplied by CLS Cell Lines Service, Eppelheim, Germany. Dulbecco’s modified eagle medium, contains 4.5 g/L D-glucose, L-glutamine, and 110 mg/L sodium pyruvate (DMEM; Invitrogen) supplemented with 2 mM L-glutamine, 10% foetal bovine serum (FBS) and penicillin-streptomycin (100 U/mL–0.1 mg/mL) was used as a culture medium (Biotech Inc., Carlsbad, CA, USA). Cells were incubated at 37 °C for 24 h with 5% CO_2_ in humidified air.

### 3.7. Cell Viability

All cells in exponential growth phase were seeded in a concentration of 1 × 10^5^ cells/mL onto the atelocollagen films with distinct concentrations of polyphenols. Cell viability was determined after 4 days in culture by MTT assay (Invitrogen Corporation). A volume of 12 mM MTT (10 μL) was taken for cell incubation performed at 37 °C for 4 h in the darkness. Thereupon, the media were decanted and washed with phosphate-buffered saline solution (PBS). The produced formazan salts were dissolved with dimethylsulphoxide (DMSO) and its concentration was measured in a spectrophotometer at 570 nm [50]. Absorbances were recorded utilising an infinite M200PRO multimode reader at 570 nm (Tecan Group, Männedorf, Switzerland), and all determinations were performed in quadruplicate. Cell morphology was qualitatively appraised every 24 h after cultivation by using an inverted phase-contrast microscope Olympus CKX 41 (Olympus, Hamburg, Germany) with an optical zoom of 100×.

### 3.8. Antimicrobial Assay *in Vitro*

The antimicrobial performance of the specimens against two pathogenic microorganisms, the gram-positive *S*. *aureus* (ATCC 6538) and the gram-negative *E*. *coli* (ATCC 8739) strains (purchased from the Czech Collection of Microorganisms, Brno, Czech Republic) was explored by agar diffusion test. Circular samples of 8 mm diameter were put into nutrient agar which was inoculated with bacteria (≈10^8^ CFU mL^−1^). After 24 h incubation at 37 °C, the diameter of the inhibition zone was measured in five directions and averaged value was calculated to evaluate the inhibition zone area. Four replicates of each substrate were tested to maintain the statistical accountability.

### 3.9. Statistical Analysis

All data were presented as the mean value ± standard deviation (SD) of each sample. Statistical comparisons were performed using Student’s t-test with a confidence level of 95% (*p* < 0.05) considered statistically significance and 99% (*p <* 0.01) considered very significant. 

## 4. Conclusions

Four edible flowers that wildly grow in the Moravian mountains were collected and their methanolic extracts were analysed by HPLC. Benzoic and hydroxycinnamic acids and flavonoids were found as the major constituents. This contribution also delved into the incorporation of these phenolic mixtures onto atelocollagen matrices. From the 16 analysed group of samples, nine were shown to be non-toxicity, with a cell viability above 80%, five samples had weak toxicity (80%–60%) and two samples displayed a moderate toxicity (<60%). Twelve samples revealed statistical significance. Antiproliferation is concomitant to concentration, relies on each extract, and all the cell viability curves followed the same pattern. Common dandelion barely affected HaCaT cell growth, while wild chive discloses more inhibition power. Cell morphology was examined by microscopy. The studied atelocollagen matrices seem to be perfectly apt for keratinocyte cell growth and proliferation. Therefore, the present approach strengthens knowledge about the use of atelocollagen, and allows consideration of these materials as potential candidates for tissue engineering and would healing applications.
